# Sickle Cell Anemia Patients in Use of Hydroxyurea: Association between Polymorphisms in Genes Encoding Metabolizing Drug Enzymes and Laboratory Parameters

**DOI:** 10.1155/2018/6105691

**Published:** 2018-01-28

**Authors:** Sètondji Cocou Modeste Alexandre Yahouédéhou, Magda Oliveira Seixas Carvalho, Rodrigo Mota Oliveira, Rayra Pereira Santiago, Caroline Conceição da Guarda, Suellen Pinheiro Carvalho, Júnia Raquel Dutra Ferreira, Milena Magalhães Aleluia, Elisângela Vitória Adorno, Marilda de Souza Gonçalves

**Affiliations:** ^1^Laboratório de Hematologia, Genética e Biologia Computacional (LHGB), Fiocruz Bahia-Instituto Gonçalo Moniz (IGM), Rua Waldemar Falcão 121, Candeal, 40296-710 Salvador, BA, Brazil; ^2^Laboratório de Pesquisa em Anemia (LPA), Departamento de Análises Clínicas, Faculdade de Farmácia, Universidade Federal da Bahia, Rua Barão do Jeremoabo 147, Ondina, 40170-115 Salvador, BA, Brazil

## Abstract

This study investigated associations between SNPs in genes encoding metabolizing drug enzymes and laboratory parameters in sickle cell anemia patients under hydroxyurea (SCA-HU^+^). We evaluated hematologic and biochemical parameters by electronic methods and SNPs by PCR-RFLP and multiplex PCR in 35 SCA-HU^+^ patients and 67 SCA-HU^−^ patients. The HbS, total cholesterol, lactate dehydrogenase, aspartate aminotransferase, total bilirubin and fractions levels, and leukocyte, eosinophil, monocyte, and erythroblast counts were reduced in SCA-HU^+^ patients (*p* < 0.05). Moreover, they presented higher HbF, C-reactive protein, and ferritin levels and elevated MCH and MCV values (*p* < 0.05). Genotype frequencies of variants GA + AA of *MPO* −463G>A and c1c2 + c2c2 of *CYP2E1* −1293G>C/−1053C>T were higher in SCA-HU^+^ patients (*p* < 0.05). Independent associations were found between the variant A allele and lower total cholesterol, between c2 allele and low alpha-1 antitrypsin and between the null GSTT1 variant and high indirect and total bilirubin in SCA-HU^+^ patients. In SCA-HU^−^ patients, independent associations were found between the variant A allele and high uric acid and between c2 allele and high urea. Our results suggest that SNPs *MPO* −463G>A, *CYP2E1* −1293G>C/−1053C>T, and *GSTT1* can be associated with alterations in lipid, inflammatory, renal, hemolytic, and hepatic profiles. However, further studies are needed to elucidate these associations.

## 1. Introduction

Sickle cell anemia (SCA) is a monogenic disease, characterized by clinical heterogeneity [1]. The clinical diversity of SCA patients has been attributed to several factors, such as sociodemographic, socioeconomic, environmental, and genetic factors [2, 3]. Fetal hemoglobin (HbF: *α*_2_*γ*_2_) is a classic genetic modulator associated with a less-severe SCA outcome, and high concentration of HbF inhibits the polymerization of the hemoglobin variant S (HbS) by formation of asymmetric hybrids with gamma (*γ*) chain and *β*^S^ chain (*α*_2_*γβ*^S^) that present high affinity for oxygen [4, 5]. Hydroxyurea (HU) is the most used drug to treat SCA patients with severe profile and increased HbF [6, 7]. Several studies have demonstrated that HU use in SCA can improve the clinical profile by reducing painful crises, hospital stay, blood transfusion, and acute chest syndrome episodes [1, 8]. Despite the HU beneficial effects, there is an interindividual variation in the treatment response and this can be due to several factors, such as environmental interaction and genetic background [4, 9, 10].

The enzymes involved in the metabolism of HU are not clearly elucidated. It was reported that monooxygenase cytochrome(s) P-450 [11, 12] and peroxidases [13, 14] can be implicated in HU metabolism. Furthermore, it is known that the efficiency of drugs depends on two phases of metabolism (phases I and II) in addition to phase III protein transporters [15–17]. In the present study, we selected enzymes of phases I (cytochrome P450: CYP450 and myeloperoxidase: MPO) and II (glutathione S-transferase: GST and NAD(P)H:quinone oxidoreductase: NQO) [15–17], since they are responsible for the metabolism of majority of available drugs and are polymorphic.

CYP2E1 is a monooxygenase highly expressed in the liver which metabolizes more than 70 endogenous and exogenous substrates including drugs, and most of these substrates are small and hydrophobic [16]. It is also implicated in the metabolism of aromatic compounds, halogenated alkanes or alkenes, benzene, and alcohols/ketones/aldehydes, and can be induced by ethanol [16]. The MPO is a heme-containing enzyme, present in high quantities in the neutrophil azurophilic granules, which catalyzes reactions of oxidation [18]. It is responsible for the production of radical species which are able to initiate lipid peroxidation. MPO activity leads to the production of hypochlorous acid (HOCl), from hydrogen peroxide and chloride, which by chlorination and/or oxidation is able to modify a wide variety of endogenous and exogenous substrates [18, 19]. NQO1, a cytosolic flavoprotein, is involved in reactions of detoxification by maintaining the antioxidant forms of intermediate metabolites produced by monooxygenases and peroxidases [20]. It is known that individuals with low NQO1 activity and high CYP2E1 activity would potentially be more susceptible to the toxic effects of xenobiotic than those with low CYP2E1 and high NQO1 activities [21]. GST(M1/T1) is involved in the detoxification of a wide variety of potentially toxic and carcinogenic electrophiles by conjugating them to glutathione [22] and enhancing xenobiotic solubility which facilitates its elimination [23].

Some genetic polymorphisms in drug-metabolizing enzymes may influence the efficiency as well as the toxicity of drugs by changing their metabolism and bioavailability [24]. Studies demonstrated the effect of some single-nucleotide polymorphisms (SNPs) present in genes *MPO*, *CYP2E1*, *NQO1*, and *GSTT1*/*GSTM1*. The c1c1 genotype of *CYP2E1* −1293G>C/−1053C>T was associated with high risk of hepatotoxicity in patients treated with antituberculosis drugs [24]. The polymorphism *MPO* −463G>A may affect the transcription of the gene, may decrease the synthesis of MPO, and has been associated with low metabolism of its substrates [19, 25]. The SNP C609T identified in *NQO* has been associated with enzyme degradation [26–28], and it was demonstrated that the loss of NQO1 activity in individuals makes it more susceptible to toxic and carcinogenic effects of reactive metabolites of quinines and a high risk of leukemia and hematotoxicity [20, 26]. Deletions in *GSTT1*/*GSTM1* have been associated with alterations in xenobiotics metabolism, as well as the patient's response to treatment [29]. Thus, based on the variability of response in SCA patients treated with HU and the effect of SNPs on the metabolism of drugs, we investigated the influence of SNPs *CYP2E1* −1293G>C/−1053C>T, *MPO* −463G>A, *NQO* C609T, and *GSTT1*/*GSTM1* on laboratory parameters in SCA patients treated with HU.

## 2. Methods

### 2.1. Subjects

The present case control study involved a total of 102 SCA patients. The case group consisted of 35 SCA patients treated with HU (SCA-HU^+^), while the control group of consisting 67 SCA patients were not treated (SCA-HU^−^). The SCA-HU^+^ patients were patients with severe clinical profiles. All SCA patients were recruited between February 2010 and July 2011 at the outpatient clinic of the Hematology and Hemotherapy Foundation of Bahia (HEMOBA). The age of the SCA-HU^+^ patients ranging between five and 42 years, with a median of 12 years, while the age of SCA-HU^−^ patients ranging between two and 55 years, with a median of 11 years. Of the SCA-HU^+^ patients, 45.71% were female, while it was 47.76% in the SCA-HU^−^ patients (Table 1). All patients reported regular use of folic acid, with SCA-HU^+^ patients taking 10 to 22 mg/kg/day. The median time of HU used in this group was about 14.5 months (range: 6–68 months).

### 2.2. Inclusion and Exclusion Criteria

All included patients had the HbSS genotype and were in a steady state, characterized as the absence of acute crisis and no use of blood therapy in the past three months. Patients on transfusion therapy, those who presented infection (HIV, HCV, HTLV I, and II, or HBV) and those who reported diseases, such as diabetes mellitus and renal or autoimmune inflammatory disease, as well as smokers and chronic alcoholics were excluded.

### 2.3. Ethical Aspects

The present study was approved by the Institutional Review Board of the Gonçalo Moniz Institute, Oswaldo Cruz Foundation (Fiocruz Bahia, Brazil), and was conducted in accordance with the Declaration of Helsinki and its revisions. Furthermore, all patients, or their legal guardians, provided a signed term of informed consent.

### 2.4. Laboratory Methods

Laboratory and molecular analyses were performed at the Clinical Analysis Laboratory, as well as the Anemia Research Laboratory, College of Pharmaceutical Sciences, at the Federal University of Bahia, in addition to the Laboratory of Hematology, Genetics and Computational Biology, at Fiocruz Bahia, Brazil.

Hematological analyses were performed using a CELL-DYN Ruby electronic cell counter (Abbott Diagnostics, Wiesbaden, Germany). Qualitative and quantitative profiles of hemoglobin (Hb) were confirmed by high-performance liquid chromatography (HPLC/Variant II; BIO-RAD, Hercules, CA, USA). Biochemical parameters were measured in serum by an immunochemistry assay using an A25 spectrophotometer autoanalyser (Biosystems SA, Barcelona, Spain). Inflammatory proteins, such as alpha-1 antitrypsin (AAT) and C-reactive protein (C-RP), were measured by immunochemistry using the Immage 800 system (Beckman Coulter, Fullerton, CA, USA). Serum ferritin was measured by immunoassay using an Access 2 Immunoassay system (Beckman Coulter, Fullerton, CA, USA).

Molecular analyses were carried out on genomic DNA extracted from peripheral blood leukocytes using the Flexigen 250 kit (Qiagen, Hilden, Germany). SNPs *CYP2E1* −1293G>C/−1053C>T (rs3813867/rs2031920), *NQO1* 609C>T (rs1800566), and *MPO* −463G>A (rs2333227) were investigated by polymerase chain reaction-restriction fragment length polymorphism (PCR-RFLP) analysis using the *Hinf*I, *Rsa*I, and *Aci*I restriction enzymes, respectively [19, 30, 31]. Deletions of *GSTT1* and *GSTM1* were investigated by multiplex PCR, and the *β*-globin gene (*HBB*) was used as an internal reaction control [22]. *β*^S^ globin gene cluster haplotypes were investigated by PCR-RFLP and talassemia *α*^2 del 3.7kb^ by PCR [32, 33].

### 2.5. Statistical Analysis

All statistical analyses were performed by EpiInfo 7.0, SPSS 17, and GraphPad Prism 5.0 software, with *p* values lower than 0.05 considered as statistically significant. Quantitative variable distributions were determined by the Shapiro-Wilk test, and mean values between two groups were compared using the unpaired *t*-test for normal distribution, while Mann–Whitney *U* test was utilized for data with nonnormal distribution. For qualitative variables, Fisher's exact test or the chi-square test (*χ*^2^ test) with Yates correction was performed. The *χ*^2^ test was used to evaluate genotypes in the case and control groups. In order to determine relative risk, odds ratios and 95% confidence intervals (95% CI) were calculated. Results were expressed as means ± standard deviation (M ± SD), numbers or percentages, where appropriate. Association analysis between laboratory parameters and polymorphisms, using a genetic dominant model, and multivariate linear regression analyses were performed to evaluate the influence of gene polymorphisms on laboratory parameters.

## 3. Results

### 3.1. Laboratory Parameters

Biochemical and hematological parameters of SCA-HU^+^ and SCA-HU^−^ patients are shown in Table 1. SCA-HU^+^ patients presented high MCV and MCH in comparison to SCA-HU^−^ patients (*p* < 0.05). HbS levels were lower in SCA-HU^+^ patients than in the control group, and HbF levels were higher in SCA-HU^+^ patients when compared to the SCA-HU^−^ patients (*p* < 0.001). Leukocyte, eosinophil, monocyte, and erythroblast counts were also lower in the SCA-HU^+^ patients (*p* < 0.05).

Lipid profile analysis showed lower concentrations of total cholesterol in SCA-HU^+^ patients than those in SCA-HU^−^ patients (*p* = 0.032). HDL-C concentration was higher in SCA-HU^+^ patients, while levels of triglycerides and LDL-C were lower in this group when compared to the SCA-HU^−^ patients. Lactate dehydrogenase (LDH), direct bilirubin (DB), indirect bilirubin (IB), and total bilirubin (TB) were reduced in the SCA-HU^+^ patients (*p* < 0.05). Aspartate aminotransferase (AST) was increased in the SCA-HU^+^ group compared to the SCA-HU^−^ group (*p* = 0.008). With regard to inflammatory markers, CRP and ferritin levels were higher in SCA-HU^+^ patients (*p* < 0.05).

### 3.2. Genotype Frequencies of *MPO* −463G>A, *NQO1* 609C>T, *CYP2E1* −1293G>C/−1053C>T, *GSTT1*, and *GSTM1* Polymorphisms as well as of Haplotypes and Thalassemia *α*

Genotype frequencies of the SNPs investigated were found to be in the Hardy-Weinberg equilibrium (*χ*^2^ test, *p* > 0.05). Genotype frequency analysis was performed in both groups of SCA patients using the dominant genetic model, with higher frequencies of GA + AA and c1c2 + c2c2 genotypes detected in SCA-HU^+^ patients as compared to SCA-HU^−^ patients. No significant difference was found when we analyzed the genotypes of *NQO1* 609C>T, *GSTT1*, and *GSTM1* polymorphisms, haplotypes, and thalassemia *α* between both groups (Tables 2 and 3).

### 3.3. Associations between Genetic Polymorphisms and Laboratory Parameters

To evaluate the influence of polymorphisms on the laboratory markers in SCA-HU^+^ patients, association analysis between laboratory parameters and polymorphisms and multivariate linear regression analyses were performed using the dominant genetic model. The same analyses were also carried out in SCA-HU^−^ patients. Only significant associations in both groups were reported (Tables 4 and 5). Association analysis showed that SCA-HU^+^ patients with the variant GA/AA genotype had low total cholesterol and LDL-C levels in comparison to those with the wild-type GG genotype (*p* < 0.05). In the SCA-HU^−^ patients, the GA/AA genotype was associated with increased concentrations of globulin, uric acid, and glucose (*p* < 0.05). The variant genotype c1c2/c2c2 was associated with reduced AAT, MCV, and MCH in the SCA-HU^+^ group (*p* < 0.05), as well as reduced monocyte counts and ferritin levels in the SCA-HU^−^ group (*p* < 0.05). Furthermore, this genotype was associated with elevated urea concentration in both groups (*p* < 0.05). The null *GSTT1* genotype was associated with elevated levels of TB and IB in SCA-HU^+^ patients (*p* < 0.05). Any significant association was observed between SNPs *NQO1* 609C>T and *GSTM1* and laboratory markers.

Multivariate analyses performed in the SCA-HU^+^ group showed that the variant GA/AA genotype was independently associated with a reduction in the total cholesterol level, whereas the variant c1c2/c2c2 genotype was found to be independently associated with reduced AAT levels. The null *GSTT1* genotype was also independently associated with increased TB and IB. In the SCA-HU^−^ group, the GA/AA genotype was independently associated with high uric acid, while the c1c2/c2c2 genotype contributed independently to increases in urea levels. No statistically significant association was found between the *NQO1* 609C>T and *GSTM1* polymorphisms and the laboratory parameters evaluated in this study.

## 4. Discussion

The present study investigated associations between SNPs *MPO* −463G>A, *CYP2E1* −1293G>C/−1053C>T, *NQO1* 609C>T, *GSTT1*, and *GSTM1* and laboratory parameters in SCA patients on HU therapy. SCA-HU^−^ patients were used as the control group. HU therapy was found to be associated with higher values of MCH and MCV, similar to what was shown by Pallis et al. [34]. On the contrary, Laurentino et al. did not observe significant differences in MCH and MCV levels among SCA-HU^+^ and SCA-HU^−^ patients [3]. The present study found an association between HU therapy and significant reductions in leukocyte, eosinophil, and monocyte counts, confirming its cytoreductive effect. This effect can improve the inflammatory profile generally observed in SCA patients, which is mainly due to increases in eosinophil and monocyte counts [34].

Our study also clearly showed significant reductions in total cholesterol concentrations among SCA-HU^+^ patients. Moreover, SCA-HU^+^ patients presented higher HDL-C levels and lower LDL-C and VLDL-C levels than SCA-HU^−^ patients, yet these values were not significant. The present results demonstrate the beneficial effect of HU on cholesterol, since it is known that plasma lipids, particularly high levels of triglycerides, are correlated with markers of intravascular hemolysis, vascular dysfunction, and pulmonary hypertension in sickle cell disease patients [35]. Recently, our research group proposed a dyslipidemic subphenotype in SCA patients [36]. Significant decreases in LDH, TB, DB, and IB were seen among SCA-HU^+^ patients, suggesting an improvement in the hemolytic profile. These findings are supported by results reported by Dehury et al., who also observed reduced levels of LDH and TB in association with HU use in HbS*β*^+^ thalassemia [37].

HU therapy was also observed to significantly reduce HbS levels, which is consistent with results by Shome et al. who also found an association between HU use and significantly reduced HbS levels in SCA patients [38]. We also detected significantly higher HbF levels in SCA-HU^+^ patients compared to controls, which confirms results in previous studies regarding the efficacy of HU in inducing HbF synthesis [3, 34, 39, 40].

SCA-HU^+^ patients exhibited high levels of the acute phase inflammatory proteins ferritin and C-RP. The elevated concentrations of ferritin seen in SCA-HU^+^ patients herein could be associated with either repeated blood transfusions or an inflammatory process in these patients. Previous studies have reported associations between frequent transfusions and elevated serum iron concentrations, which subsequently lead to increased ferritin concentrations [41, 42]. The first hypothesis seems far less probable, since all patients who received transfusions three months prior to blood sample collection were excluded. Hence, the second hypothesis appears more probable, suggesting that, despite the cytoreductive effect of HU at doses lower than its maximum tolerated dose (MTD), therapy does not reduce levels of C-RP, which is produced by the liver in response to proinflammatory cytokines and is considered a plasma biomarker for low-grade systemic inflammation [43, 44].

Two phases of drug metabolism (phases I and II) have been described in the literature [15–17]. HU metabolism has been reported to occur via monooxygenase cytochrome(s) P-450 [11, 12] and peroxidases [13, 14]. However, the underlying metabolic pathway remains to be fully elucidated. Since CYP2E1 and MPO, which are phase I enzymes, and GST(T1/M1) and NQO1, phase II enzymes [15–17], are highly implicated in the metabolism of the majority of endogenous and exogenous substrates (antimetabolite drugs, alcohol, benzene, and isoniazid), these enzymes were selected for study, in addition to some associated SNPs which were chosen due to their importance for the activity of the enzymes. SNP *CYP2E1* −1293G>C/−1053C>T affects genomic transcriptional activity [45]. SNP *MPO* −463G>A results in the loss of a binding site for transcription factor SP1, leading to decreased gene expression [18]. The deletion of *GST(T1/M1)* leads to loss of GST activity [46]. SNP *NQO1* 609C>T decreases NQO1 activity [47, 48].

In the present study, we observed high frequencies of the GA + AA and c1c2 + c2c2 genotypes in SCA-HU^+^ patients in comparison with SCA-HU^−^ patients. The GA/AA genotype was found to be associated with low total cholesterol and LDL-C levels in SCA-HU^+^ patients, as well as with high glucose levels in SCA-HU^−^ patients. A recent study conducted in patients with diabetes mellitus also demonstrated an association between the GA/AA genotype and reduced levels of total cholesterol, in addition to elevated glucose levels [19]. Thus, our findings confirm the association between the variant A allele and levels of cholesterol and glucose, especially in SCA-HU^+^ patients since, we found that (1) HU is associated with reduction of low total cholesterol levels and (2) SCA-HU^+^ patients with the variant A allele have significant reduction in total cholesterol and LDL-C levels compared with those carrying the wild-type G allele. Furthermore, the GA/AA genotype was found to be associated with significant increases in uric acid and globulin in SCA-HU^−^ patients. However, this association was not seen in SCA-HU^+^ patients, which suggests that HU is able to reduce the levels of these parameters in SCA patients carrying these genotypes. Interestingly, we observed that the GA/AA genotype was independently associated with low concentrations of total cholesterol in SCA-HU^+^ patients and with high concentrations of uric acid in SCA-HU^−^ patients.

The c1c2/c2c2 genotype which affects the transcriptional activity of CYP2E1 was associated with a significant reduction of the RBC index (MCV and MCH) in SCA-HU^+^ patients, suggesting that the variant allele c2 compromises the beneficial effect of HU to increase the RBC index, as seen in the present study and shown by Pallis et al. [34]. This reduction of the RBC index can represent a risk factor for the occurrence of vasoocclusive events in SCA patients with the c1c2/c2c2 genotype even using HU, since it is known that a low MCV value is associated with an increase of the blood viscosity [8]. An independent association was found between the c1c2/c2c2 genotype and reduced levels of AAT in SCA-HU^+^ patients. Hassan et al. reported the crucial role played by CYP2E1 in the repression of hepatic antioxidant parameters and therefore in the onset and propagation of Isoniazid-induced liver damage, mainly in the inflammatory response induced by LPS [49]. Piao et al. observed a high variant allele c2 frequency in patients with alcoholic or nonalcoholic fatty liver and then suggested that the variant allele may be important for the pathogenesis of fatty liver [50]. Therefore, our findings suggest that SCA-HU^+^ patients with the c2 allele are susceptible to producing and/or accumulating metabolites of HU, due to lack of functional enzyme, which can induce an inflammatory reaction with production of anti-inflammatory protein, and then might lead to hepatic damage. The c1c2/c2c2 genotype was associated with high urea levels in SCA-HU^+^ patients. In the multivariate analysis performed in SCA-HU^−^ patients, this association between the c1c2/c2c2 genotype and high level of urea was statistically significant and independent. As seen in the present study, the c2 allele was associated with low AAT as well as high urea in the SCA-HU^+^ patients suggesting that the variant allele can be associated with the initiation of renal dysfunction since a recent study performed by Maicas et al. showed that the treatment with human AAT in the murine model improved acute renal dysfunction reducing significantly urea levels, which demonstrate the role of AAT in kidney disease [51]. In the SCA-HU^−^ patients, the c1c2/c2c2 genotype was also associated with reduced monocyte counts and ferritin levels. However, the mechanism explaining this association is not clearly understood.

It is known that the null *GSTT1* genotype leads to the lack of the enzyme and that GST has a pivotal role in cellular protection against the cytotoxic effects caused by xenobiotic and drug [52]. In addition, GST is more expressed in hepatocytes, and apart from its catalytic function in detoxification, the members of the M and T classes of GSTs have the capacity to bind a nonsubstrate ligand (bilirubin) allowing its intracellular transport [53]. So, the independent association found between the null *GSTT1* genotype and bilirubin (TB and IB) levels in SCA-HU^+^ patients demonstrates the persistence of hemolytic anemia despite HU use or hepatic injury, which is probably associated with the presence of toxic metabolites derived from HU. In addition, recent works reported existence of association between high TB, hemolytic anemia, and hepatic dysfunction [54, 55]. Therefore, we can suggest that the wild *GSTT1* genotype is important for the improvement of hemolysis or reduction of hepatic injury in SCA-HU^+^ patients. In contrast, a previous study conducted by Muslu et al. in newborns with pathogenic jaundice did not observe a statistically significant difference in TB levels between patients with the null *GSTT1* genotype and those with the wild *GSTT1* genotype [53]. However, in this study, the authors suggest that the null *GSTM1* genotype may affect ligandin functions in hepatocytes, which are important in bilirubin transportation and therefore, patients with the null *GSTM1* genotype might have elevated levels of bilirubin [53]. Abdel Ghany et al. also demonstrated that the null *GSTM1* variant represents a higher risk of developing hiperbilirubinemia in neonates [56]. However, in the present study, no statistically significant association was found between null the *GSTM1* variant and bilirubin levels. The SNP *NQO* 609C>T did not also show any significant association with the laboratory parameters evaluated in the present study.

## 5. Conclusion

To the best of our knowledge, this is the first study that investigated the association between SNPs in genes encoding enzymes that metabolize drugs and laboratory parameters among SCA patients on HU therapy. The results revealed that HU increases HbF levels and the RBC index, reduces leukocyte counts, aspartate aminotransferase, and bilirubin levels as described in the literature and observed in the present study. It also seems to be associated with changes in lipid profile. Furthermore, at a dose under MTD, HU does not reduce the acute phase inflammatory protein levels. The variants A allele and c2 allele and the null *GSTT1* genotype seem to be associated with alterations in lipid, inflammatory, renal, hemolytic, and hepatic profiles, improving or compromising the treatment of the SCA patients under HU, depending on the SNP and parameter affected. The principal limitation of the present study is the enrollment of patients with age ranging between 2 and 55 years since some laboratory parameters are influenced by age. However, further studies are needed to explore and better understand the mechanistic basis of the associations found in the present study.

## Figures and Tables

**Table 1:** Laboratory profiles of SCA patients treated (SCA-HU^+^) and untreated (SCA-HU^−^) with HU.

	SCA-HU^+^ patients (*N* = 35)	SCA-HU^−^ patients (*N* = 67)	
	Mean ± SD	Mean ± SD	*p* value
Age (years)	12 (5–42)	11 (2–55)	0.05^∗^
Gender			
Male, *N* (%)	19 (54.29%)	35 (52.24%)	**—**
Female, *N* (%)	16 (45.71%)	32 (47.76%)	
Hemoglobins			
HbS (%)	79.38 ± 10.34	85.92 ± 5.79	**<0.001** ^∗∗^
HbF (%)	13.66 ± 6.25	9.82 ± 5.84	**0.003** ^∗∗^
Hemolysis			
RBC (×10^9^/mL)	2.57 ± 0.39	2.63 ± 0.41	0.498^∗^
Hemoglobin (g/dL)	8.57 ± 1.28	8.15 ± 1.17	0.096^∗^
Reticulocyte (%)	6.79 ± 2.25	6.93 ± 2.22	0.813^∗^
Hematocrit (%)	24.87 ± 3.82	23.50 ± 3.19	0.057^∗^
MCH (pg)	32.54 ± 3.69	30.97 ± 3.31	**0.032** ^∗^
MCHC (%)	34.51 ± 1.33	34.89 ± 1.41	0.200^∗^
MCV (fL)	94.13 ± 10.08	89.20 ± 7.38	**0.006** ^∗^
Erythroblast (/10^2^ leukocytes)	1.46 ± 1.33	2.47 ± 2.25	**0.048** ^∗∗^
Haptoglobin (mg/dL)	5.83 ± 0.00	5.84 ± 0.05	0.485^∗^
Hemolysis plus hepatic			
Aspartate aminotransferase (U/L)	45.34 ± 14.97	54.45 ± 16.56	**0.008** ^∗^
Total bilirubin (mg/dL)	1.83 ± 0.72	2.73 ± 1.36	**<0.001** ^∗∗^
Indirect bilirubin (mg/dL)	1.35 ± 0.67	2.07 ± 1.32	**0.003** ^∗∗^
Direct bilirubin (mg/dL)	0.48 ± 0.16	0.63 ± 0.26	**0.009** ^∗∗^
Iron serum (mcg/dL)	102.50 ± 45.80	105.70 ± 49.81	0.770^∗∗^
Lactate dehydrogenase (U/L)	947.20 ± 354.80	1169.00 ± 457.20	**0.023** ^∗∗^
Platelets			
Platelet (×10^3^/mL)	431.50 ± 153.50	483.50 ± 133.20	0.082^∗∗^
Leukocytes			
Leukocyte (/mL)	12016.14 ± 4778.30	13490.30 ± 3319.20	**0.047** ^∗∗^
Eosinophil (/mL)	429.80 ± 280.20	971.40 ± 861.60	**<0.001** ^∗∗^
Monocyte (/mL)	840.90 ± 466.40	999.70 ± 354.70	**0.009** ^∗∗^
Lipid and glycemic			
Total cholesterol (mg/dL)	118.20 ± 16.06	127.40 ± 21.12	**0.032** ^∗^
HDL-C (mg/dL)	33.00 ± 7.47	31.34 ± 7.57	0.244^∗∗^
LDL-C (mg/dL)	70.05 ± 20.78	75.36 ± 18.78	0.199^∗^
VLDL-C (mg/dL)	17.64 ± 6.16	20.03 ± 10.05	0.596^∗∗^
Triglycerides (mg/dL)	88.61 ± 31.19	100.40 ± 49.93	0.609^∗∗^
Glucose (mg/dL)	74.23 ± 7.40	72.34 ± 7.86	0.243^∗^
Renal			
Urea (mg/dL)	16.86 ± 5.21	15.66 ± 5.31	0.282^∗^
Creatinine (mg/dL)	0.47 ± 0.16	0.41 ± 0.13	0.067^∗∗^
Hepatic			
Alanine aminotransferase (U/L)	20.74 ± 8.49	24.59 ± 13.79	0.309^∗∗^
Total protein (g/dL)	8.17 ± 0.42	7.97 ± 0.92	0.223^∗^
Albumin (g/dL)	4.47 ± 0.39	4.45 ± 0.33	0.821^∗^
Globulin (g/dL)	3.70 ± 0.56	3.51 ± 0.88	0.261^∗^
Inflammation			
Uric acid (mg/dL)	4.15 ± 1.22	4.05 ± 1.14	0.677^∗^
C-reactive protein (mg/L)	8.26 ± 6.40	4.95 ± 3.87	**0.003** ^∗∗^
Ferritin (ng/dL)	308.50 ± 219.60	155.7 ± 95.41	**<0.001** ^∗∗^
Alpha-1 antitrypsin (mg/dL)	161.30 ± 41.01	153.60 ± 34.86	0.328^∗^

RBC: red blood cell; MCH: mean corpuscular hemoglobin; MCV: mean corpuscular volume; MCHC: mean corpuscular hemoglobin concentration; HbS: S hemoglobin; HbF: fetal hemoglobin; HDL-C: high-density lipoprotein cholesterol; LDL-C: low-density lipoprotein cholesterol; VLDL-C: very low-density lipoprotein cholesterol; SCA-HU^+^: SCA patient treated with HU; SCA-HU^−^: SCA patient untreated with HU; ^∗^unpaired *t*-test, ^∗∗^Mann Whitney *U* test.

**Table 2:** Genotype frequencies of *MPO −463G>A*, *NQO1 609C>T*, *CYP2E1 −1293G>C/−1053C>T*, *GSTT1*, and *GSTM1* polymorphisms using the dominant genetic model in SCA patients treated (SCA-HU^+^) and untreated (SCA-HU^−^) with HU.

Genotypes	SCA-HU^+^ patients *N* (%)	SCA-HU^−^ patients *N* (%)	OR (95% CI)	*p* value
*MPO −463G>A*	*N* = 35 (100%)	*N* = 66 (100%)		
GG	10 (28.57%)	34 (51.52%)	2.66	**0.045** ^∗^
GA + AA	25 (71.43%)	32 (48.48%)	(1.10–6.39)	
*NQO1 609C>T*	*N* = 35 (100%)	*N* = 67 (100%)		
CC	24 (68.57%)	41 (61.19%)	0.72	0.604^∗^
CT + TT	11 (31.43%)	26 (38.81%)	(0.30–1.72)	
*CYP2E1 −1293G>C/−1053C>T*	*N* = 35 (100%)	*N* = 67 (100%)		
c1c1	26 (74.29%)	63 (94.03%)	5.45	**0.009** ^∗∗^
c1c2 + c2c2	9 (25.71%)	4 (5.97%)	(1.54–19.28)	
*GSTT1*	*N* = 35 (100%)	*N* = 67 (100%)		
*GSTT1*+	26 (74.29%)	49 (73.13%)	0.94	0.911^∗^
*GSTT1−*	9 (25.71%)	18 (26.87%)	(0.37–2.39)	
*GSTM1*	*N* = 35 (100%)	*N* = 67 (100%)		
*GSTM1*+	21 (60%)	45 (67.16%)	1.36	0.616^∗^
*GSTM1−*	14 (40%)	22 (32.84%)	(0.58–3.18)	

SCA-HU^+^: SCA patient treated with HU; SCA-HU^−^: SCA patient untreated with HU; *MPO*: myeloperoxidase; *CYP2E1*: cytochrome P450 2E1; *GSTT1/GSTM1*: glutathione S-transferase T1/M1; *NQO1*: NADP(H) quinone oxidoreductase; *N*: number; ^∗^corrected Yates, ^∗∗^exact Fisher test; OR: odds ratio; 95% CI: 95% confidence interval.

**Table 3:** Genotype frequencies of *α*-thalassemia and allelic frequencies of haplotype in SCA patients treated (SCA-HU^+^) and untreated (SCA-HU^−^) with HU.

	SCA-HU^+^ patients	SCA-HU^−^ patients	
Tal *α*^2del 3.7kb^	*N* = 27	*N* = 65	*p* value
*αα*/*αα*	23 (85.19%)	51 (78.46%)	0.571^∗∗^
−*α*/*αα*	4 (14.81%)	14 (21.54%)	
−−/*αα*	0 (0%)	0 (0%)	

Haplotype/chromosome	*N* = 68	*N* = 112	*p* value
CAR	28 (41.18%)	61 (50%)	—
BEN	36 (52.94%)	51 (41.80%)	
ATIP	4 (5.88%)	10 (8.20%)	

SCA-HU^+^: SCA patient treated with HU; SCA-HU^−^: SCA patient untreated with HU; *N*: number; CAR: Bantu; BEN: Benin; ATIP: atypical. ^∗∗^Exact Fischer test.

**(a):** 

Parameters	SCA-HU^+^ patients		
	*MPO* (GA + AA) *N* = 25	*MPO* (GG) *N* = 10	*p* value
Total cholesterol (mg/dL)	115.8 ± 15.37	139.8 ± 29.98	**0.004** ^∗^
LDL-C (mg/dL)	64.92 ± 16.96	84.31 ± 24.62	**0.014** ^∗^

	*CYP2E1* (c1c2 + c2c2) *N* = 9	*CYP2E1* (c1c1) *N* = 26	*p* value
Alpha-1 antitrypsin (mg/dL)	137.16 ± 31.95	169.99 ± 40.92	**0.037** ^∗^
Urea (mg/dL)	19.78 ± 5.99	15.85 ± 4.62	**0.049** ^∗^
MCH (rg)	30.10 ± 2.99	33.38 ± 3.17	**0.019** ^∗^
MCV (%)	88.12 ± 11.80	96.24 ± 8.72	**0.036** ^∗^

	*GSTT1* ^−^ *N* = 9	*GSTT1* ^+^ *N* = 26	*p* value
Total bilirubin (mg/dL)	2.62 ± 1.33	1.71 ± 0.71	**0.043** ^∗∗^
Indirect bilirubin (mg/dL)	2.09 ± 1.38	1.25 ± 0.67	**0.045** ^∗∗^

**(b):** 

Parameters	SCA-HU^−^ patients		
	*MPO* (GA + AA) *N* = 32	*MPO* (GG) *N* = 34	*p* value
Globulin (g/dL)	3.74 ± 0.95	3.30 ± 0.78	**0.043** ^∗^
Uric acid (mg/dL)	4.76 ± 1.55	3.67 ± 0.97	**0.001** ^∗^
Glucose (mg/dL)	74.66 ± 6.60	70.21 ± 8.52	**0.020** ^∗^

	*CYP2E1* (c1c2 + c2c2) *N* = 4	*CYP2E1* (c1c1) *N* = 63	*p* value
Monocytes (/mL)	685 ± 175	1020 ± 355	**0.048** ^∗∗^
Ferritin (ng/dL)	57.18 ± 17.99	210.0 ± 176.4	**0.002** ^∗∗^
Urea (mg/dL)	35.25 ± 29.67	16.67 ± 9.24	**0.048** ^∗∗^

LDL-C: low-density lipoprotein cholesterol; MCH: mean corpuscular hemoglobin; MCV: mean corpuscular volume; *MPO*: myeloperoxidase; *CYP2E1*: cytochrome P450 2E1; *GSTT1*: glutathione S-transferase 1; SCA-HU^+^: SCA patient treated with HU; SCA-HU^−^: SCA patient untreated with HU. ^∗^Unpaired *t*-test; ^∗∗^Mann Whitney *U* test.

**(a):** 

	SCA-HU^+^ patients				
Independent variables	Dependent variable	*β*	*p* value	*R* ^2^	*p* value of the model
Model					
*MPO*^∗^	Total cholesterol	−0.391	**0.001**	0.744	**<0.001** ^a^
MCH		−0274	**0.011**		
LDL-C		0.667	**<0.001**		
Iron serum		0.266	**0.013**		
Lactate dehydrogenase		−0.272	**0.019**		

Model					
*CYP2E1*^∗^	Alpha-1 antitrypsin	−0.350	**0.017**	0.596	**<0.001** ^a^
Uric acid		−0.563	**0.001**		
Urea		0.270	**0.047**		
Creatinine		0.386	**0.014**		
Iron serum		−0398	**0.005**		
Direct bilirubin		0.440	**0.007**		

Model					
*GSTT1*^∗^	Total bilirubin	0.464	**0.004**	0.283	**0.005** ^a^
Creatinine		−0.337	**0.033**		

Model					
*GSTT1*^∗^	Indirect bilirubin	0.433	**0.009**	0.233	**0.014** ^a^
Creatinine		−0.286	0.077		

**(b):** 

	SCA-HU^−^ patients				
Independent variables	Dependent variable	*β*	*p* value	*R* ^2^	*p* value of model
Model					
*MPO*^∗^	Uric acid	0.379	**0.001**	0.244	**<0.001** ^a^
Total cholesterol		0.293	**0.010**		

Model					
*CYP2E1*^∗^	Urea	0.389	**0.001**	0.175	**0.007** ^a^
Globulin		−0.187	0.124		
Uric acid		0.110	0.363		

LDL-C: low-density lipoprotein cholesterol; MCH: mean corpuscular hemoglobin; *MPO*: myeloperoxidase; *CYP2E1*: cytochrome P450 2E1; *GSTT1*: glutathione S-transferase; SCA-HU^+^: SCA patient treated with HU; SCA-HU^−^: SCA patient untreated with HU; *R*^2^: coefficient of determination; *β*: coefficient of regression; ^a^ANOVA; ^∗^dominant genetic model.
